# Is *N*-Hacking Ever OK? The consequences of collecting more data in pursuit of statistical significance

**DOI:** 10.1371/journal.pbio.3002345

**Published:** 2023-11-01

**Authors:** Pamela Reinagel

**Affiliations:** Department of Neurobiology, School of Biological Science, University of California San Diego, La Jolla, California, United States of America

## Abstract

Upon completion of an experiment, if a trend is observed that is “not quite significant,” it can be tempting to collect more data in an effort to achieve statistical significance. Such sample augmentation or “*N*-hacking” is condemned because it can lead to an excess of false positives, which can reduce the reproducibility of results. However, the scenarios used to prove this rule tend to be unrealistic, assuming the addition of unlimited extra samples to achieve statistical significance, or doing so when results are not even close to significant; an unlikely situation for most experiments involving patient samples, cultured cells, or live animals. If we were to examine some more realistic scenarios, could there be any situations where *N*-hacking might be an acceptable practice? This Essay aims to address this question, using simulations to demonstrate how *N*-hacking causes false positives and to investigate whether this increase is still relevant when using parameters based on real-life experimental settings.

## Abstract 2

When an experiment comes to an end, it can be tempting to collect more data if a trend is observed that is “not quite significant”. This Essay uses simulations to investigate if this type of sample augmentation can ever be an acceptable practice and, if so, when it could be beneficial.

## Introduction

There has been much concern in recent years about the lack of reproducibility of results in some scientific fields, leading to a call for improved statistical practices [1–5]. The recognition of a need for better education in statistics and greater transparency in reporting is justified and welcome, but rules and procedures should not be applied by rote without comprehension. Experiments often require substantial financial resources, scientific talent, and the use of finite and precious resources; there is therefore an ethical imperative to use these resources efficiently. Thus, to ensure both the reproducibility and efficiency of research, experimentalists need to understand the underlying statistical principles behind the rules.

One rule of null hypothesis significance testing is that if a sample size *N* is chosen in advance, it may not be changed (augmented) after seeing the results [1,6–9]. In my experience, this rule is not well known among biologists and is commonly violated. Many researchers engage in “*N*-hacking”: incrementally adding more observations to an experiment when a preliminary result is “almost significant.” Indeed, it is not uncommon for reviewers of manuscripts to require that authors collect more data to support a claim if the presented data do not reach significance. Prohibitions against collecting additional data are therefore met with considerable resistance and confusion by the research community.

So, what is the problem with *N*-hacking? What effects does it have on the reliability of a study’s results and are there any scenarios where its use might be acceptable? In this Essay, I aim to address these questions using simulations representing different experimental scenarios (Box 1) and discuss the implications of the results for experimental biologists. I am not claiming or attempting to overturn any established statistical principles; yet, although there is nothing theoretically new here, the numerical results may be surprising, even for those familiar with the theoretical principles at play.

**Box 1. Simulation details**

The specific sampling heuristic simulated in this Essay is meant to be descriptive of practice and is different in details from established formal adaptive sampling methods [6,10–12]. The simulations can be taken to represent a large number of independent studies, each collecting separate samples to test a different hypothesis. All simulations were performed in MATLAB 2018a. Definitions of all terms and symbols are summarized in S1 Appendix. The MATLAB code for all these simulations and more can be found in [13], along with the complete numeric results of all computationally intensive simulations.

## The simulations

### What effect does *N*-hacking have on the false positive rate?

The first task is to establish the effect that *N*-hacking has on the false positive rate. To do this, experiments were simulated by comparing 2 independent samples of size *N* drawn from the same normal distribution. An independent sample Student’s *t* test was used to reject or fail to reject the null hypothesis that the samples came from distributions with the same mean, with the significance threshold *p*<0.05. Because the samples always came from the same distribution, any positive result will be a false positive. I call the observed false positive rate when the null hypothesis is true *FP*_0_ (“FP null”), also known as the type I error rate, to emphasize that this is not the same as “the probability a positive result is false” (False Positive Risk). By construction, in this scenario the *t* test produces false positives at a rate of exactly *α*, the significance threshold (0.05 in this case).

**Fig 1:**
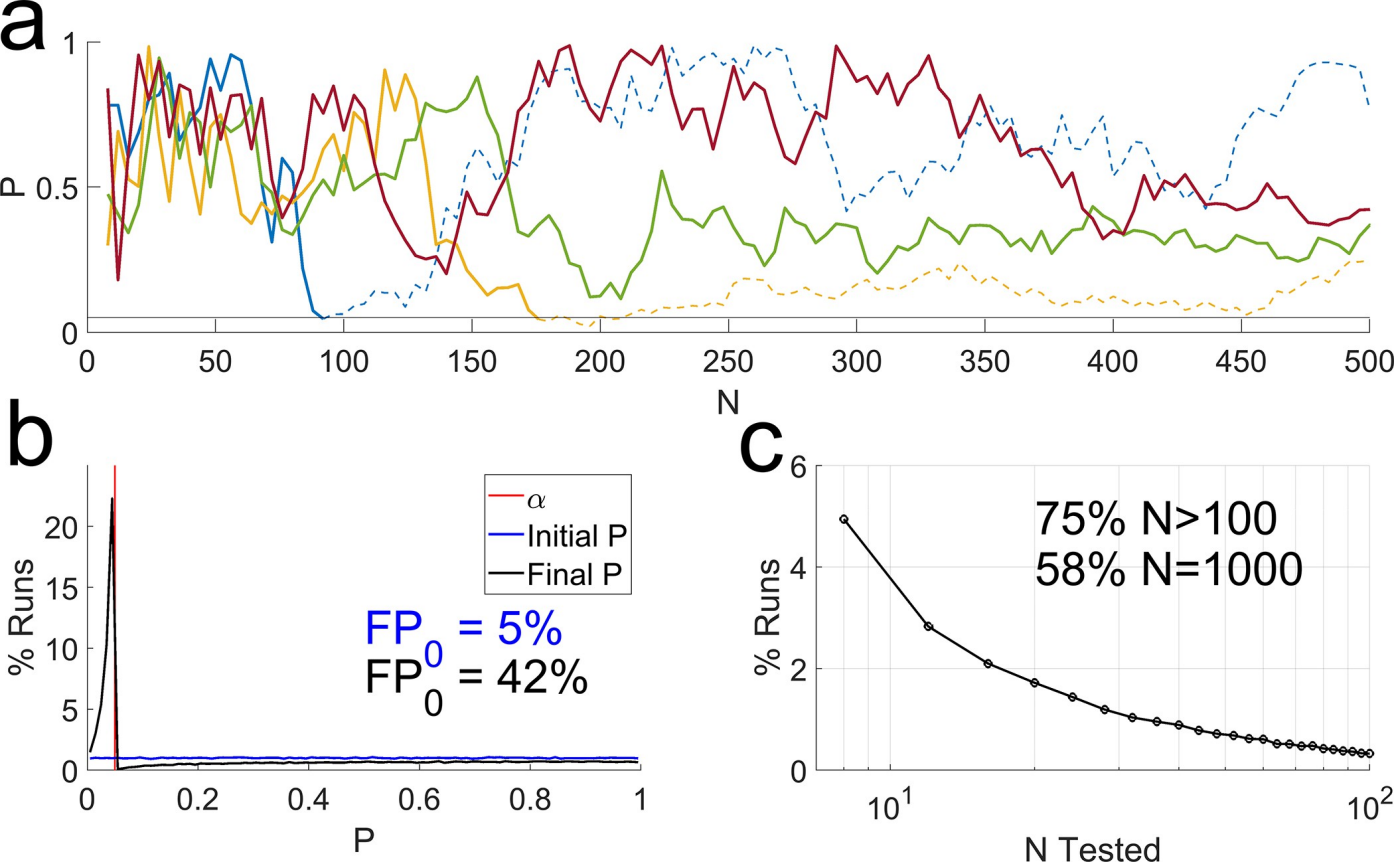
The problem with *N*-hacking. Simulation of experiments in which there was no true effect, starting with samples of size *N* = 8. If the result was nonsignificant, we added 4 more and retested, until either the result was significant or *N* = 1,000. (**a**) Evolution of “*p* values” of 4 simulated experiments, as *N* was increased. If sampling were terminated when *p*<*α* (solid blue and gold curves), this would produce false positives. If sampling had continued, those would have become nonsignificant again (dashed blue and gold curves). (**b**) Distribution of initial and final “*p* values” of 10^5^ such experiments, in bins of width 0.01. Vertical red line indicates the nominal *α* (0.05). FP_0_ values indicate the false positive rates associated with the same colored curves (integral from *p* = 0 to *p* = *α*). (c) Distribution of final sample sizes, based on counts of each discrete sample size. The fraction of runs that exceeded *N* = 100 or that reached *N* = 1,000 are indicated.

However, if researchers continue collecting more data until they get a significant effect, some true negatives will be converted to false positives. For example, suppose many separate labs each ran a study with sample size *N* = 8, where in every case, there was no true effect to be found. If all used a criterion of *α* = 0.05, we expect 5% to obtain false positive results. But suppose all the labs with “nonsignificant” outcomes responded by adding 4 more data points to their sample and testing again, repeating this as necessary until either the result was significant, or the sample size reached *N* = 1,000. The interim “*p* values” would fluctuate randomly as the sample sizes grew (Fig 1A) and, in some cases, the “*p* value” would cross the significance threshold by chance. If these studies ended as soon as *p*<*α* and reported significant effects, these would represent excess false positives, above and beyond the 5% they intended to accept.

In one simulation of 10,000 such experiments, there were 495 false positives (5%) in the initial *t* test, but 4,262 false positives (43%) after *N*-hacking (Fig 1B). Therefore, the final “*p* values” after *N*-hacking are not valid *p* values—they do not reflect the probability of observing a difference at least this large by chance if there were no real effect. This has been pointed out by many others [1,6–9] and serves to illustrate why *N*-hacking can be problematic for users of *p* values.

However, this scenario postulates unrealistically industrious and stubborn researchers. Suppose the experimental units used were mice. For the 5% of labs that obtained a false positive at the outset, the sample size was a reasonable *N* = 8 mice. All other labs had larger final samples. Three quarters of the simulated labs would have tested over 100 mice, and over half of the simulated labs tested 1,000 mice before giving up (Fig 1C). Moreover, in 75% of the simulated runs, additional data were collected after observing an interim “*p* value” in excess of 0.9. These choices are frankly implausible.

Suppose instead that the sample size would be increased only if *p*<0.10, and only up to a maximum of *N* = 32 mice; this strict upper limit on the sample size reflects the fact that real experiments have finite resources. In this constrained *N*-increasing procedure, *p* values falling within the eligible window (0.05≤*p*<0.10) are treated as inconclusive outcomes or can be viewed as defining a “promising zone” of the negative results most likely to be false negatives. These inconclusive/promising cases are resolved by collecting additional data. Experiments with interim *p* values falling above the upper limit are considered futile and abandoned.

**Fig 2:**
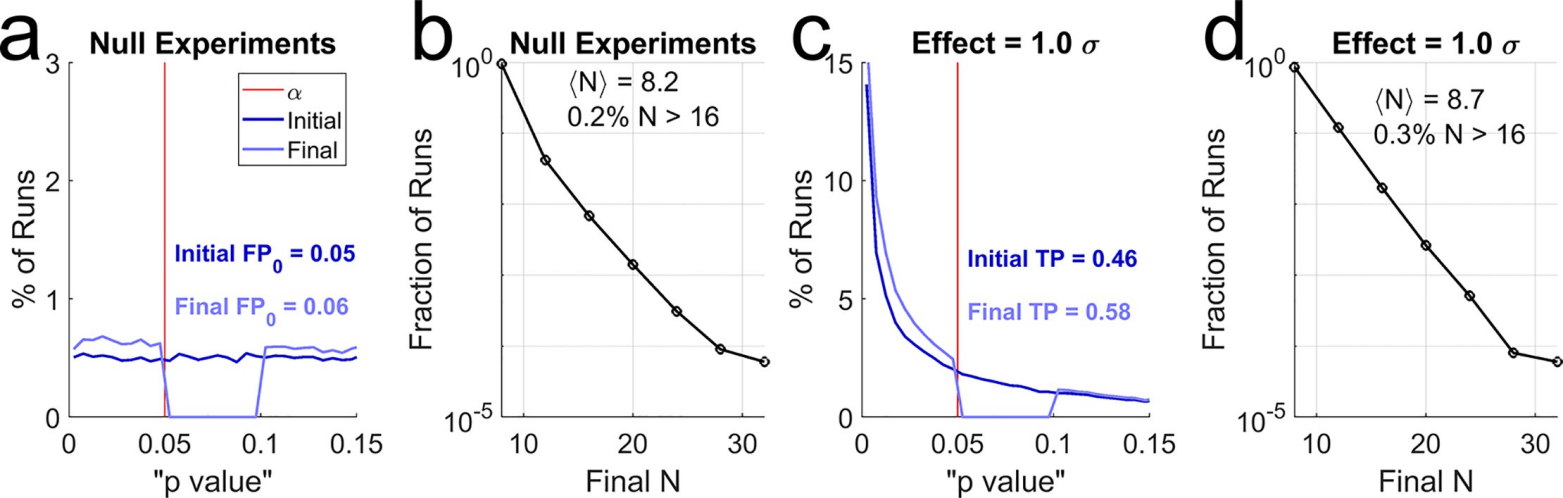
Constrained sample augmentation. Hypothetical sampling procedure in which an initial sample of *N* = 8 is incremented by 4, only if 0.05≤*p*<0.10, up to a maximum of *N* = 32. (a) Distribution of initial *p* values (dark blue) vs. final “*p* values” (pale blue) in simulations with no real effect. Horizontal scale is expanded in the region around *α* (red line) to show detail. Note the depletion of “*p* values” in the eligibility window (trough in pale curve). “FP_0_” indicates the false positive rate before (Initial) vs. after (Final) augmentation. In this simulation of 10^5^ runs, the observed false positive rate of this procedure was *FP*_0_ = 0.0625. (b) Distribution of final sample sizes in the simulations shown in (a). 〈*N*〉 indicates the mean final sample size; the percentage of runs exceeding *N* = 16 is also shown. Note that the sample cap of *N* = 32 was rarely reached. (c) Distribution of initial and final “*p* values” for the same sampling policies as (a) and (b), when all experiments had a real effect of size 1 standard deviation. “TP” indicates the observed true positive rate before and after augmentation. (d) Distribution of final sample sizes of experiments in (c). Mean sample size and percent exceeding *N* = 16 are also shown.

This constrained version of *N*-hacking (more neutrally, “sample augmentation”) also yielded an increase in the rate of false positives, but this effect was rather modest, yielding a false positive rate *FP*_0_ = 0.0625 instead of the intended 0.05 (Fig 2A). Note that no correction for multiple comparisons was applied. On average, following this procedure resulted in a negligible increase in the sample size and only rarely resulted in more than twice the initially planned sample (Fig 2B). Therefore, if a researcher routinely collected additional data to shore up almost-significant effects, constrained by a conservative cutoff for being “almost” significant, the inflation of false positives would be inconsequential. These 2 extreme examples (Fig 1 versus Fig 2) show that, from the point of view of the false positive rate *FP*_0_, sample augmentation can either be disastrous or benign depending on the details of the decision rule. I will return to the question of what parameters are compatible with reasonably limited false positive rates in a later section.

In addition to false positives, however, we must also consider the effect on false negatives. To this end, I repeated the simulations assuming a true effect of 1 standard deviation (SD) difference in the means. In these simulations, every positive is a true positive, and every negative is a false negative. In the initial samples of *N* = 8, the real effect was detected in some but not all experiments (Fig 2C, dark blue curve). The initial true positive rate, 46%, is simply the statistical power for a fixed sample size of *N* = 8 per group to detect a 1 SD effect.

Notably, constrained sample augmentation increased the statistical power (Fig 2C, pale blue curve) while only slightly increasing the average final sample (Fig 2D). A fixed-*N* experiment using the augmentation procedure’s type I error rate for *α* (*α* = 0.0625) and *N* = 9 has less power than the augmented procedure (56% versus 58%). Thus, constrained sample augmentation can increase the chance of discovering real effects, even compared to fixed-*N* experiments with the same false positive rate and an equal or larger final sample size. From this perspective, constrained *N*-hacking represents a net benefit.

### How does *N*-hacking affect the positive predictive value?

What most biologists really care about is whether their positive results will be reliable in the sense of identifying real effects. This is not given by 1−*p* or 1−*α*, as many erroneously believe, but another quantity called the positive predictive value (PPV; Box 2). To determine PPV, one must also know the effect size and the fraction of experiments in which a real effect exists—the prior probability, or prior for short. In any real-world experiment, the true effect size and prior are unknown. But in simulations we stipulate these values, so the PPV is well defined and can be numerically estimated.

**Box 2. Positive predictive value**

**Fig 3:**
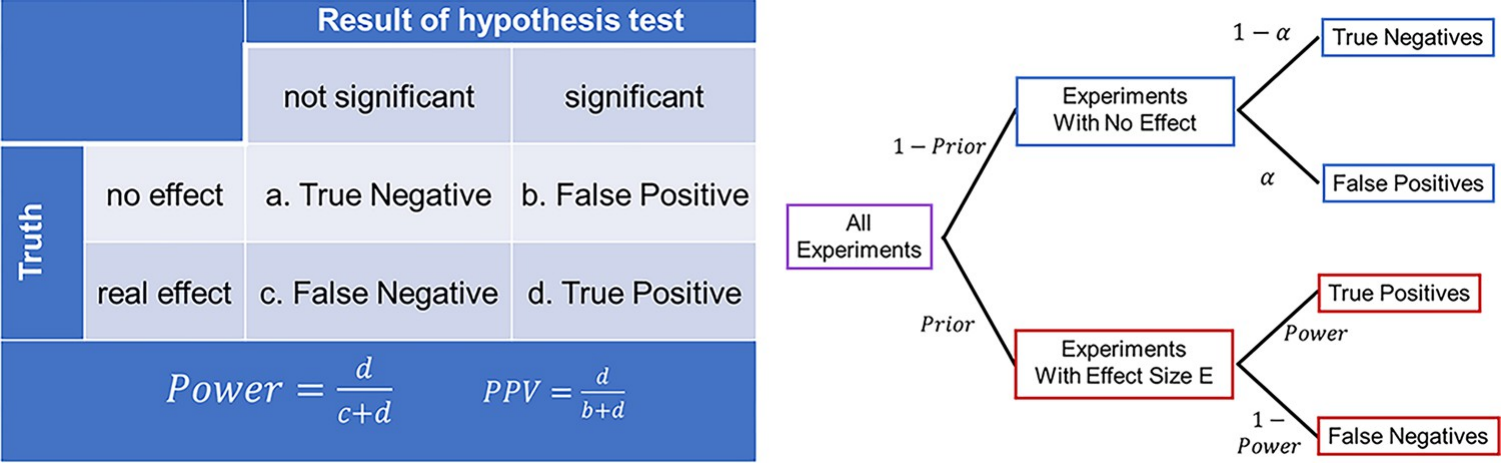
Schematic overview of hypothesis testing and PPV.

A scientific community tests many hypotheses. The effect for which any experiment is testing is either absent or present (Fig 3; Truth, rows in table). The outcome of a binary significance test is either negative or positive (Fig 3; Result, columns of table). This yields 4 types of outcomes: true negatives (a); false positives (type I errors, b); false negatives (type II errors, c); and true positives (d). The statistical power of a procedure is defined as the fraction of real effects that yield significant effects. The PPV of a procedure is defined as the fraction of significant effects that are real effects. The tree diagram illustrates how these quantities are related. The probability of a false positive when there is no real effect depends only on the procedure *α* (Fig 3; blue boxes, upper right). The probability of a true positive when there is a real effect depends on the power (Fig 3; red boxes, lower right), which in turn depends on both *α* and the effect size E. The probability that a significant event is real (the PPV) further depends on the fraction of all experiments that are on the red versus the blue branch of this tree (the prior). In the real world, effect sizes and priors are not known. For a more in-depth primer, see [14].

To illustrate how sample augmentation impacts PPV, simulations were carried out exactly as described in the previous section, but now 10% of all experiments had a real effect (1*σ*, as in Fig 2C and 2D), and the remaining 90% had no real effect (as in Fig 2A and 2B). In this toy world, the point of doing an experiment would be to find out if a particular case belongs to the null group or the real effect group. The PPV is defined as the fraction of all positive results that are true positives.

**Fig 4:**
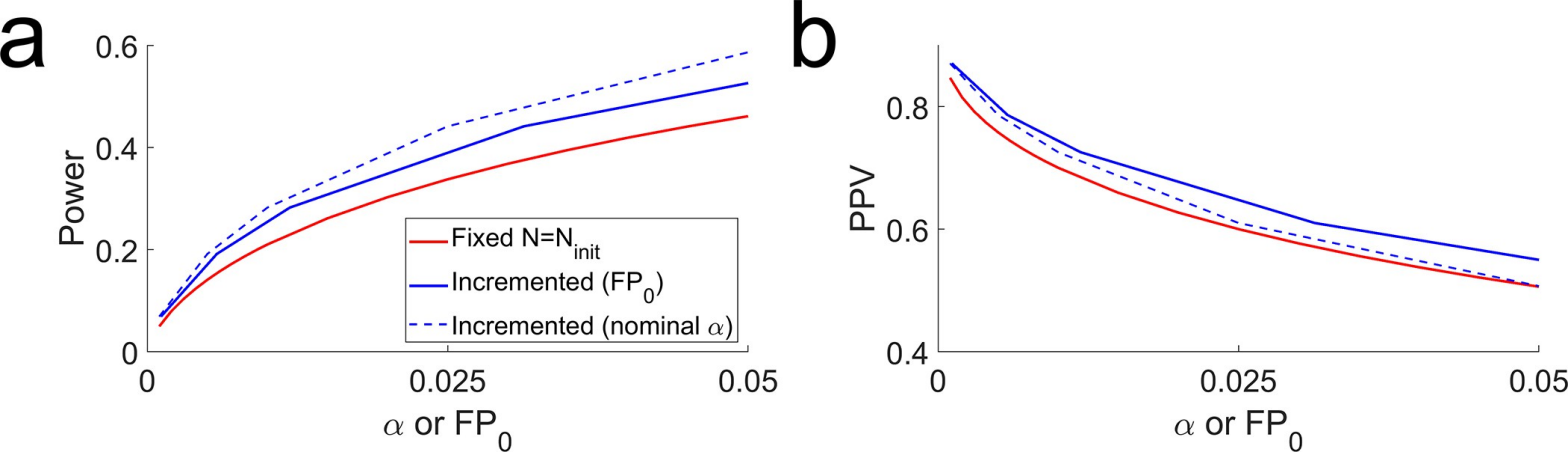
Constrained augmentation can increase both power and PPV. Simulations in which 10% of all experiments had a real effect (*Pr* = 0.1) of size 1 standard deviation (*E* = 1*σ*), varying the significance criterion *α*. (a) Statistical power of the fixed-*N* procedure with *N* = 8 (red), compared to constrained augmentation with *N*_*init*_ = 8, *N*_*incr*_ = 4, *N*_*max*_ = 32, *w* = 1 (blue). For the sample augmenting procedure, results are plotted as a function of the observed false positive rate *FP*_0_ (solid blue) or the nominal criterion *α* (dashed blue). (b) PPV for the same simulations analyzed in (a). Statistical power and PPV were computed analytically for the fixed-*N* procedure or estimated from *M* = 10^4^/*α* simulated experiments for the incrementing procedure.

Constrained sample augmentation can increase both power and PPV. For example, using *N* = 8 and *α* = 0.01, statistical power increased from 21% before to 28% after augmentation; PPV increased from 70% to 73%. These effects depend quantitatively on *α*, which can be shown by simulating the sampling procedure of Fig 2 for several choices of *α* (Fig 4). The average final sample size 〈*N*_final_〉 ranged from 8.02 (for *α* = 0.001) to 8.28 (for *α* = 0.05). Therefore, performance of constrained augmentation can be reasonably compared to the fixed-*N* procedure with *N* = 8 (Fig 4, red curves). The sample augmenting procedure had higher power than fixed-*N*, even after correcting for the false positive rate of the procedure (Fig 4A, solid blue curve), and yielded higher PPV than fixed-*N*, whether or not the false positive rate of the procedure was corrected for (Fig 4B). Overall, although unplanned sample augmentation or *N*-hacking is widely considered a “questionable research practice,” under these conditions it would yield results at least as reliable as those obtained by sticking with the originally planned sample size, if not more reliable.

### How do the parameters used affect the consequences of *N*-hacking?

#### Dependence of *FP*_0_ on parameters

Given that unconstrained sample augmentation can drastically increase false positives (Fig 1), whereas under some conditions constrained sample augmentation only negligibly increases false positives (Fig 2), it would be useful to have a general rule for what false positive rate to expect for any arbitrary constraint condition. This would be quite difficult to derive analytically, but can easily be explored using numerical simulations.

The critical factor for the false positive rate (*FP*_0_) is the width of the window of *p* values that are eligible for augmentation, relative to the significance criterion *α*. To express this, we can define the variable *w* as the width of the eligibility window in units of *α*. For example, in the case of Fig 2A, *α* = 0.05 and *w* = 1, such that one would reject the null hypothesis (declare a significant effect) if *p*<0.05, fail to reject the null (no significant effect) if *p*≥0.10, and add observations for the inconclusive *p* values in between. In the egregious *N*-hacking case simulated in Fig 1, *α* = 0.05 and *w* = 19, such that one would reject the null hypothesis if *p*<0.05, fail to reject if *p*>1.00, and increment otherwise. For a table of the lower and upper boundary *p* values defining the inconclusive/promising window for different choices of *w*, see S1 Table. In the rest of this section, I call the initial sample size of an experiment *N*_*init*_, the number of observations added between re-tests the sample increment *N*_*incr*_, and the maximum sample size one would test *N*_*max*_. A table of these and other variable definitions is provided in S1 Appendix.

Before discussing simulation results, we can develop an intuition. The false positive rate after sample augmentation cannot be less than *α*, because this many false positives are obtained when the initial sample is tested. Subsequent sample augmentation can only add to the false positives. Furthermore, the false positive rate cannot exceed the upper cutoff *p* value of *α*(1+*w*), because all experiments with initial *p* values above this are immediately abandoned as futile. Exactly *wα* experiments are deemed inconclusive or promising (eligible for additional data collection), so no more than this many can be converted to false positives. Indeed, no more than half of them should be converted, because if there is no true effect, collecting additional data is more likely to shift the observed effect towards the null than away from it.

**Fig 5:**
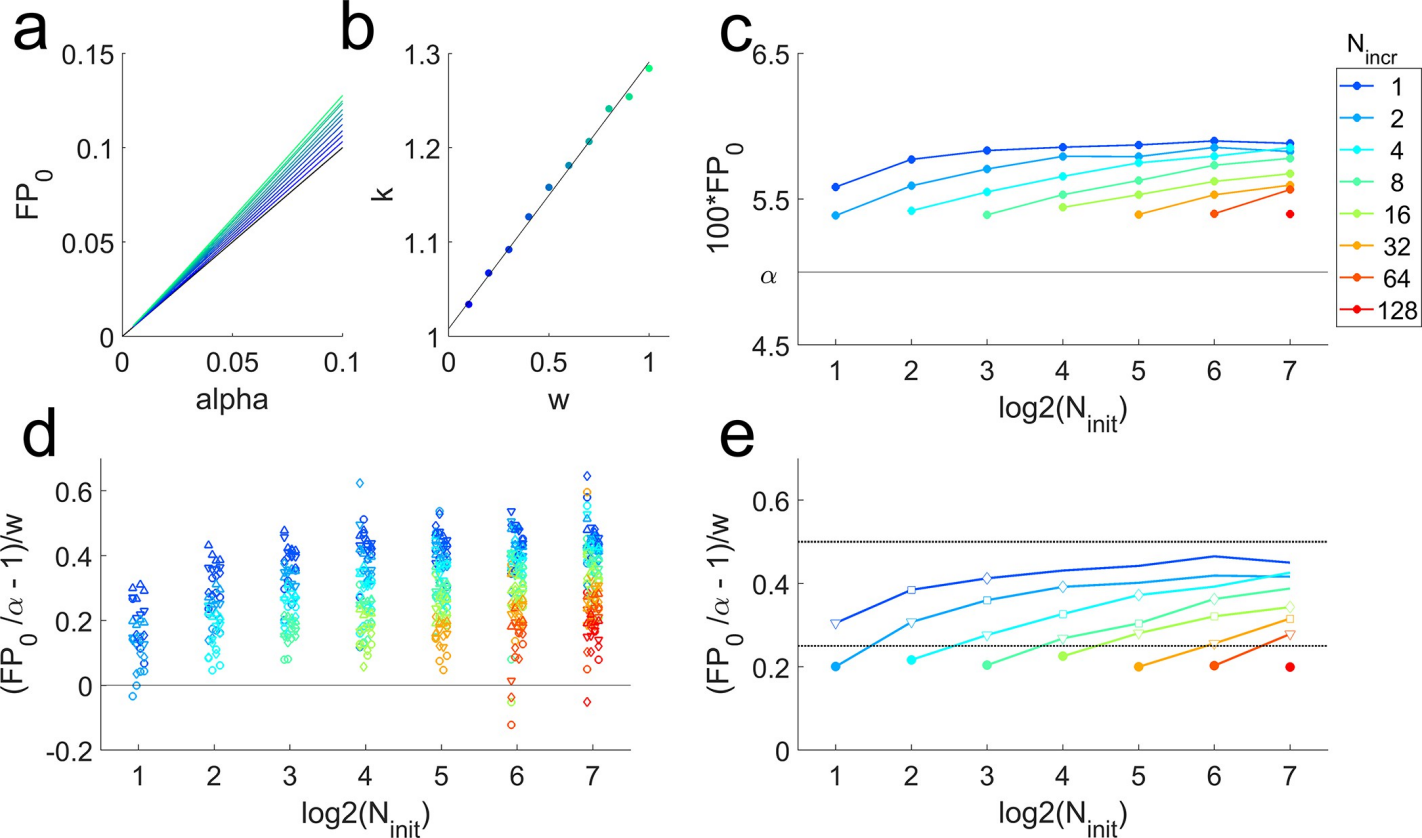
Dependence of false positive rate on sample augmentation parameters. Simulations of constrained sample augmentation when the null hypothesis is true, using *N*_*init*_ = 12, *N*_*incr*_ = 6, *N*_*max*_ = 24, *M* = 10^6^ simulated experiments per condition. (a) The observed false positive rate (*FP*_0_) vs. *α*. Color indicates *w* (cf. panel b). For each *w*, *FP*_0_ is plotted for each simulated value of *α* [0.005, 0.01, 0.025, 0.05, 0.1], and the data points connected. The identity line (black), *FP*_0_ = *α*, is the false positive rate of the standard Fixed-*N* procedure. (b) The slopes *k* obtained from linear fits to the data shown in (a), plotted as a function of window size *w* (colored symbols). The dependence of the slope *k* on *w* is not linear in general, but is approximately linear in this parameter range (linear fit, black). (c) The realized *FP*_0_ of the constrained *N*-increasing procedure, as a function of log_2_ *Ni*_*nit*_ (horizontal axis) and *N*_*incr*_(colors), for the case *α* = 0.05, *w* = 0.4, *N*_*max*_ = 256. The *FP*_0_ is always elevated compared to *α* (black line), but this is more severe when the intial sample size is larger (curves slope upward) or the incremental sample growth is smaller (cooler colors are higher). Color key at right applies to panels (c–e). (d) Results for 4 choices of *α* (0.005, 0.010, 0.025, or 0.050; symbol shapes) and *w* (0.1, 0.2, 0.3, or 0.4; small horizontal shifts), plotted as $(\frac{FP}{\alpha }-1)/w$ (vertical axis) to reveal regularities. For the fixed-*N* procedure *FP*_0_ = *α*, so this equation reduces to 0 (black line). Positive values on this scale indicate an increase in the false positive rate compared to the fixed-*N* procedure. (e) Summary of simulations in (d) obtained by fitting the equation *FP* = (*cw*+1)*α*, as in panel (b). Symbols indicate simulations in which *N*_*incr*_ = *N*_*init*_ (closed circles), *N*_*incr*_ = *N*_*init*_/2 (open triangles), *N*_*incr*_ = *N*_*init*_/4 (open squares), and *N*_*incr*_ = *N*_*init*_/8 (open diamonds). Upper dashed black line is a proposed empirical bound ${FP}_{0}<\alpha (1+\frac{w}{2})$. Lower black line is a proposed bound for *N*_*incr*_ = *N*_*init*_, ${FP}_{0}<\alpha (1+\frac{w}{4})$.

To show this numerically, I simulated a range of choices a range of choices of both *w* and α using *N*_*init*_ = 12, *N*_*incr*_ = 6, *N*_*max*_ = 24 (Fig 5). I focused on what I consider realistic choices of *α* (not exceeding 0.1) and *w* (not exceeding 1). In this range, simulations show that for any given choice of *w*, the false positive rate depends linearly on *α* (Fig 5A). The slopes of these lines are in turn an increasing function of the decision window *w* (Fig 5B, symbols). For small *w*, this relationship is approximately linear.

The false positive rate also depends on the initial sample size and the increment size. To illustrate this, I repeated these simulations for *N*_*init*_ ranging from 2 to 128 initial sample points and increments *N*_*incr*_ ranging from 1 to *N*_*init*_, capping the maximum total sample size at *N*_*max*_ = 256_._ The false positive rate was inflated more severely when the intial sample size was larger or the incremental sample growth step smaller (Fig 5C). The increment cannot get any smaller than *N*_*incr*_ = 1, and this curve has leveled off by *N*_*init*_ = 256, so we can take *N*_*init*_ = 256, *N*_*incr*_ = 1 (observed *FP*_0_ = 0.059) as the worst case scenario for this choice of *α* and *w*. In the explored regime, the maximum sample size was rarely reached and therefore had little influence on overall performance characteristics.

The false positive rate is a systematic function of *α* and *w*. Because the false positive rate scales linearly with *α* (Fig 5A) and approximately linear with *w* over this range of values for *w* (Fig 5B), results of the simulations for all combinations of *α* and *w* can be summarized on one plot by linearly scaling them (Fig 5D). This confirms that the false positive rate is bounded (upper dashed line, Fig 5E), as expected from the intuition given above. When the increment step is the same as the initial sample size, there appears to be a lower bound (lower dashed line, Fig 5E). Simulations up to *w* = 0.4 are shown, but these empirically justified bounds are not violated when *w* is larger because the dependence on *w* is sublinear, such that the normalized false positive rate decreases slightly as *w* increases. Of course the absolute false positive rate still increases with *w*. In the egregious *N*-hacking case of Fig 1 (*α* = 0.05, *w* = 19), for example, the empirical bound yields a not-very-comforting bound of *FP*_0_<0.52 (still a conservative estimate relative to the numerically estimated value, *FP*_0_ = 0.42). In summary, *N*-hacking does increase the false positive rate, but by a predictable and small amount in some realistic scenarios. Regardless of the initial sample size *N*, if *p* is less than twice the significance threshold, one can collect more data in batches of *N*/2 or *N* at a time and still keep the false positive rate in check.

#### Dependence of PPV on parameters

**Fig 6:**
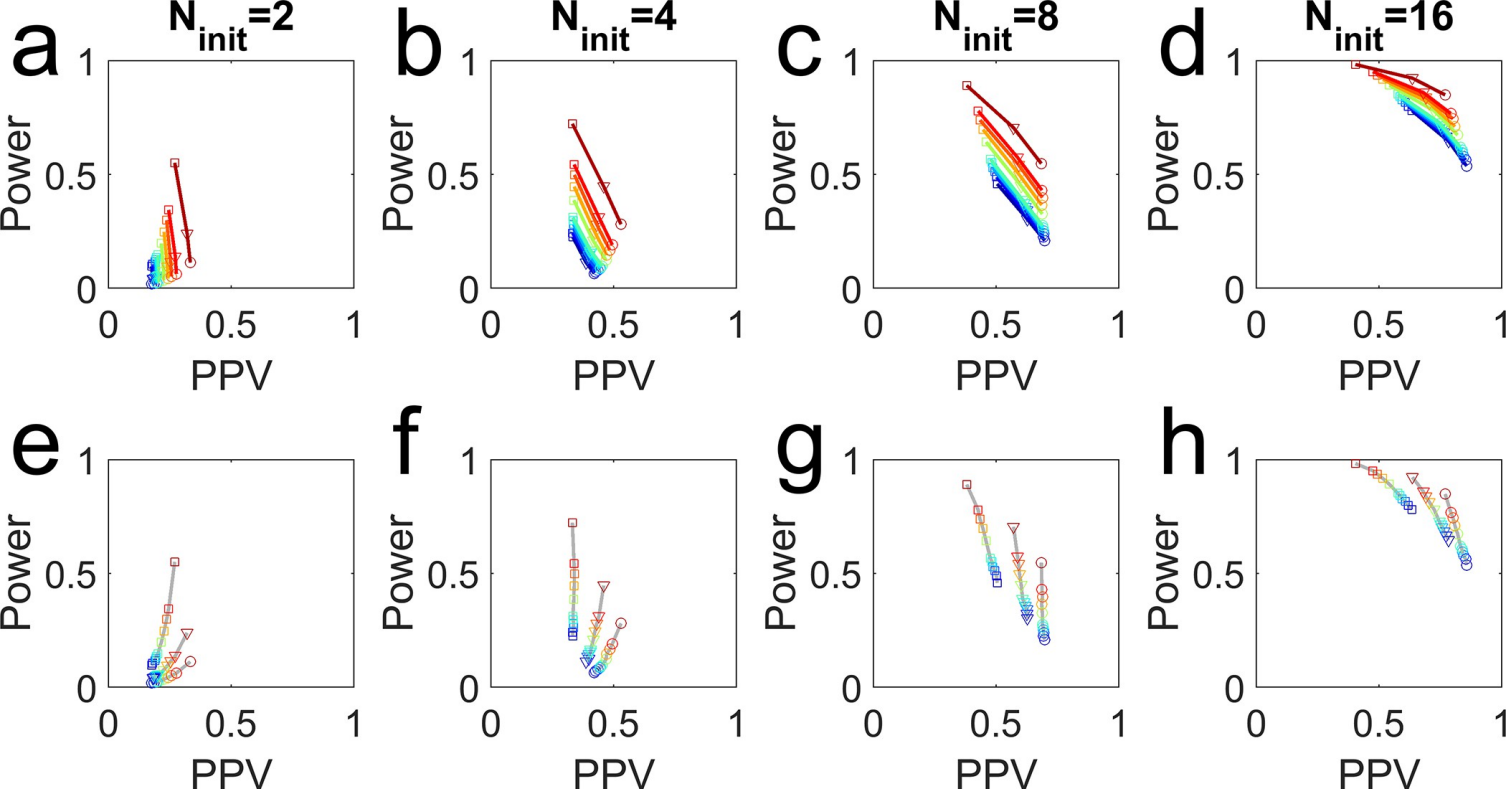
Uncorrected sample augmentation improves the PPV–power trade-off. Plots show the measured PPV vs. statistical power in simulations with effect size *E* = 1*σ* and prior effect probability *p*(*H*_1_) = 0.10, with *N*_*init*_ as indicated on column title, *N*_*incr*_ = 1, *N*_*max*_ = 50. Each symbol represents the results from *M* = 10^6^ simulated experiments, with no corrections. Symbols indicate *α* (○ = 0.01, ▽ = 0.02, □ = 0.05). Colors indicate *w* (blue➔red = 0, 0.2, 0.4, 0.6, 0.8, 1, 2, 3, 4, 5, 10). Note that dark blue (*w* = 0) is the fixed-*N* procedure. **Top panels:** simulations with the same *w* and different *α* are connected with curves. **Bottom panels:** the same data, but simulations with same *α* and different *w* are connected with gray curves.

In the section on how *N*-hacking affects the PPV, I demonstrated one condition in which uncorrected sample augmentation improved both statistical power and PPV, but this is not always the case. To illustrate this, I repeated simulations like those in Fig 4 out to extreme choices of *w* (0.2 to 10), using a worst-case increment size (*N*_*incr*_ = 1) and a liberal sampling cap (*N*_*max*_ = 50), again varying *α*. The initial sample size was varied from extremely underpowered (*N*_*init*_ = 2) to appropriately powered (*N*_*init*_ = 16) for the fixed-*N* procedure (Fig 6).

For a fixed choice of *α*, increasing *w* (more aggressive sample augmentation) always increases statistical power (Fig 6 bottom row, warm colors are above cool colors along any gray curve). This makes sense: The more freely one would collect a few more data points, the more often false negatives will be rescued to true positives. However, this only sometimes increases PPV compared to the fixed-*N* procedure. For example, for *N*_*init*_ = 4, *α* = 0.01, PPV increases with *w* (Fig 6 bottom left, circles: gray curve slope is positive) but for *N*_*init*_ = 8, *α* = 0.05, PPV decreases with increasing *w* (Fig 6 bottom right, squares: gray curve slope is negative).

Nevertheless, uncorrected sample augmentation always produces a higher PPV and power than the fixed-*N* procedure with the same false positive rate; or a higher PPV and lower false positive rate than the fixed-*N* procedure with the same statistical power (see S2 Table for examples). For any sample-augmentation parameters (Fig 6, curves other than dark blue in top panels), if we find the point along the dark blue curve (fixed-*N*) that has the same power, the PPV is lower; or if we find the point on the fixed-*N* curve with the same PPV, the power is lower. Curves with higher *w* lie strictly above and to the right those of lower *w*, including fixed-*N* (*w* = 0). In this sense, *N*-hacking is always better than not *N*-hacking.

## Implications of the simulation results

Many researchers are unaware that it matters when or how they decide how much data to collect when testing for an effect. The first take home message from this Essay is that if you are reporting *p* values, it does matter. Increasing the sample size after obtaining a nonsignificant *p* value will on average lead to a higher rate of false positives, if the null hypothesis is true. This has been said many times before, but most authors warn that this practice will lead to extremely high false positive rates [6–9]. This certainly can occur, if a researcher were to increment their sample size no matter how far from *α* the *p* value was and continue to collect data until *N* was quite large (Fig 1). But I have personally never met an experimental biologist who would do that.

If extra data were only collected if the *p* value were quite close to *α*, then the effects on the false positive rate would be modest and bounded. The magnitude of the increase in the false positive rate depends quantitatively on the initial sample size (*N*_*init*_), the significance criterion (*α*), the promising zone or eligibility window (*w*), and the increment size (*N*_*incr*_). In the previous section, I provide an intuitive explanation and empirical validation for an upper bound on the false positive rate. Moreover, sample augmentation strictly increases the PPV achievable for any given statistical power compared to studies that strictly adhere to the initially planned *N*; an outcome that remained true for both underpowered and well-powered regimes. To my knowledge, this particular sampling procedure has not been considered before, but the basic principles underlying the benefits of adaptive sampling have long been known in the field of statistics [15].

In the literature, optional stopping of an experiment or *N*-hacking has often been flagged as an important cause of irreproducible results. But in some regimes, uncorrected data-dependent sample augmentation could increase both statistical power and PPV relative to a fixed-*N* procedure of the same nominal *α*. Therefore, in research fields that operate in that restricted regime, it is simply not true that *N*-hacking would lead to an increased risk of unreproducible results. A verdict of “statistical significance” reached in this manner is if anything more likely to be reproducible than results reached by fixed-*N* experiments with the same sample size, even if no correction is applied for sequential sampling or multiple comparisons. Therefore, if any research field operating in that parameter regime has a high rate of false claims, other factors are likely to be responsible.

### Some caveats

I have asserted that certain practices are common based on my experience, but I have not done an empirical study to support this claim. Moreover, I have simulated only one “questionable” practice: post hoc sample augmentation based on an interim *p* value. I have seen this done to rescue a nonsignificant result, as simulated here, but I have also seen it done to verify a barely significant one (a practice which results in *FP*_0_<*α*). In other contexts, I suspect researchers flexibly decide when to stop collecting data on the basis of directly observed results or visual inspection of plots, without interim statistical tests. Such decisions may take into account additional factors such as the absolute effect size, a heuristic which could have even more favorable performance characteristics [16]. From a metascience perspective, a comprehensive study of how researchers make sampling decisions in different disciplines (biological or otherwise), coupled with an analysis of how the observed operating heuristics would impact reproducibility, would be quite interesting [17].

In this Essay, I have discussed the effect of *N*-hacking on type I errors (false positives) and type II errors (false negatives). Statistical procedures may also be evaluated for errors in effect size estimation: type M (magnitude) and type S (sign) errors [18]. Even in a fixed-*N* experiment, effect sizes estimated from “significant” results are systematically overestimated. This bias can be quite large when *N* is small. This concern also applies to the low-*N* experiments described here, but sample augmentation does not increase either the type M or type S error compared to fixed-*N* experiments [13].

### What about batch effects?

It is often necessary to collect data in batches and/or over long time periods for pragmatic reasons. Differences between batches or over time can be substantial sources of variability, even when using a fixed-*N* procedure. Therefore, one should check if there are batch or time-varying effects and account for them in the analysis if necessary. This is not unique to *N*-hacking, but with incremental sample augmentation this concern will always be applicable. Likewise, if the experimental design is hierarchical, a hierarchical model is needed, regardless of sampling procedure [19].

I have simulated a balanced experimental design, with the same *N* in both groups in the initial batch, with the sample size of both groups being augmented equally in each sample augmentation step. This is recommended, especially in multi-factorial designs with many groups, as it minimizes the risk of confounding batch effects with the effects under study. Moreover, selectively augmenting the sample size in some groups but not others can introduce other confounds and interpretation complexities [20].

## So, is *N*-hacking ever OK?

Researchers today are being told that if they have obtained a nonsignificant finding with a *p* value just above *α*, it would be a “questionable research practice” or even a breach of scientific ethics to add more observations to their data set to improve statistical power. Nor may they describe the result as “almost” or “bordering on” significant. They must either run a completely independent larger-*N* replication or fail to reject the null hypothesis. Unfortunately, in the current publishing climate, this generally means relegation to the file drawer. Depending on the context, there may be better options.

In the following discussion, I use the term “confirmatory” to mean a study designed for a null hypothesis significance test, intended to detect effects supported by *p* values or “statistical significance”. I use the term “non-confirmatory” as an umbrella term to refer to all other kinds of empirical research. While some have used the term “exploratory” for this meaning [21–23], their definitions vary, and the word “exploratory” already has other specific meanings in this context [24,25], making this terminology more confusing than helpful [26,27].

An ideal confirmatory study would completely prespecify the sample size or sampling plan and every other aspect of the study design, and furthermore, establish that all null model assumptions are exactly true and all potential confounds are avoided or accounted for. This ideal is unattainable in practice. Therefore, real confirmatory studies fall along a continuum from very closely approaching this ideal, to looser approximations.

A very high bar is appropriate when a confirmatory experiment is intended to be the sole or primary basis of a high-stakes decision, such as a clinical trial to determine if a drug should be approved. At this end of the continuum, the confirmatory study should be as close to the ideal as humanly possible, and public preregistration is reasonably required. The “*p* value” obtained after unplanned incremental sampling is not a valid *p* value, because without a prespecified sampling plan, you can never truly know or prove what you would have done if the data had been otherwise, so there is no way to know how often a false positive would have been found by chance. *N*-hacking forfeits control of the type I error rate, whether the false positive rate is increased or decreased thereby. Therefore, in a strictly confirmatory study, *N*-hacking is not OK.

That being said, planned incremental sampling is not *N*-hacking. There are many established adaptive sampling procedures that allow flexibility in when to stop collecting data, while still producing rigorous *p* values. These methods are widely used in clinical trials, where costs, as well as stakes, are very high. It is beyond the present scope to review these methods, but see [6,10–12] for more information. Simpler, or more convenient, prespecified adaptive sampling schemes are also valid, even if they are not optimal [8]. In this spirit, the sampling heuristic I simulated could be followed as a formal procedure (S2 Appendix).

A less-perfect confirmatory study is often sufficient in lower-stakes conditions, such as when results are intended only to inform decisions about subsequent experiments, and where claims are understood as contributing to a larger body of evidence for a conclusion. In this research context, transparent *N*-hacking in a mostly prespecified study might be OK. Although data-dependent sample augmentation will prevent determination of an exact *p* value, the researchers may still be able to estimate or bound the *p* value (see S2 Appendix). When such a correction is small and well justified, this imperfection might be on a par with others we routinely accept, such as assumptions of the statistical test that cannot be confirmed or which are only approximately true.

In my opinion, it is acceptable to report a *p* value in this situation, as long as there is full disclosure. The report should state that unplanned sample augmentation occurred, report the interim *N* and *p* values, describe the basis of the decision as honestly as possible, and provide and justify the authors’ best or most conservative estimate of the *p* value. With complete transparency (including publication of the raw data), readers of the study can decide what interpretation of the data is most appropriate for their purposes, including relying only on the initial, strictly confirmatory *p* value, if that standard is most appropriate for the decision they need to make.

However, many high-quality research studies are mostly or entirely non-confirmatory, even if they follow a tightly focused trajectory or are hypothesis (theory) driven. For example, “exploratory experimentation” aims to describe empirical regularities prior to formulation of any theory [25]. Development of a mechanistic or causal model may proceed through a large number of small (low-power) experiments [28,29], often entailing many “micro-replications” [30]. In this type of research, putative effects are routinely re-tested in follow-up experiments or confirmed by independent means [31–34]. Flexibility may be essential to efficient discovery in such research, but the interim decisions about data collection or other aspects of experimental design may be too numerous, qualitative, or implicit to model. In this kind of research, the use of *p* values is entirely inappropriate; however, this does not mean abandoning statistical analysis or quantitative rigor. Non-confirmatory studies can use other statistical tools, including exploratory data analysis [24] and Bayesian statistics [35]. Unplanned sample augmentation is specifically problematic for *p* values; other statistical measures do not have the same problem (for an example, compare Fig 1 to S1 Fig) [36,37]. Therefore, in transparently non-confirmatory research, unplanned sample augmentation is not even *N*-hacking. If a sampling decision heuristic of the sort simulated here were employed, researchers would not need to worry about producing an avalanche of false findings in the literature.

A common problem in biology is that many non-confirmatory studies report performative *p* values and make “statistical significance” claims, not realizing that this implies and requires prospective study design. It is always improper to present a study as being prospectively designed when it was not. To improve transparency, authors should label non-confirmatory research as such, and be able to do so with no stigma attached. Journals and referees should not demand reporting of *p* values or “statistical significance” in such studies, and authors should refuse to provide them. Where to draw the boundary between approximately confirmatory and non-confirmatory research remains blurry. My own opinion is that it is better to err on the side of classifying research non-confirmatory, and reserve null hypothesis significance tests and *p* values for cases where there is a specific reason a confirmatory test is required.

## Conclusions

In this Essay, I used simulations to demonstrate how *N*-hacking can cause false positives and showed that, in a parameter regime relevant for many experiments, the increase in false positives is actually quite modest. Moreover, results obtained using such moderate sample augmentation have a higher PPV than non-incremented experiments of the same sample size and statistical power. In other words, adding a few more observations to shore up a nearly significant result can increase the reproducibility of results. For strictly confirmatory experiments, *N*-hacking is not acceptable, but many experiments are non-confirmatory, and for these, unplanned sample augmentation with reasonable decision rules would not be likely to cause rampant irreproducibility.

In the pursuit of improving the reliability of science, we should question “questionable” research practices, rather than merely denounce them [38–47]. We should also distinguish practices that are inevitably severely misleading [48–50] from ones that are only a problem under specific conditions, or that have only minor ill effects. A quantitative, contextual exploration of the consequences of a research practice is more instructive for researchers than issuing a blanket injunction. Such thoughtful engagement can lead to more useful suggestions for improved practice of science or may reveal that the goals and constraints of the research are other than what was assumed.

## Supporting information

S1 Table Relation of the window width parameter w to the lower and upper cutoff *p* values defining the eligibility window, for the case of *α* = 0.05. (PDF)

S2 Table *N*-hacking compared with alternative fixed-*N* policies. (PDF)

S1 Fig Data from the simulations shown in Fig 1, re-analyzed using log likelihood ratios instead of *p* values. (PDF)

S1 Appendix Definitions of terms and variables as used in this paper. (PDF)

S2 Appendix A conservative bound on type I error rate. (PDF)
