# Supplementary material for: Is N-Hacking Ever OK? The consequences of collecting more data in pursuit of statistical significance
Source: PLoS Biol. 2023 Nov 1;21(11):e3002345. doi: 10.1371/journal.pbio.3002345 (PMC10619921; doi:10.1371/journal.pbio.3002345)
Supplement: S1 Table — (PDF) [file pbio.3002345.s001.pdf]

$\alpha = 0.05$			
w	P_{min}	P_{max}	$\alpha_{procedure} \leq$
0	0.05	0.050	0.050
0.2	0.05	0.060	0.055
0.5	0.05	0.075	0.063
0.8	0.05	0.088	0.069
1	0.05	0.100	0.075
2	0.05	0.150	0.100
18	0.05	0.950	0.500
19	0.05	1.00	0.520

S1 Table. Relation of the window width parameter *w* to the lower and upper cutoff *p* values defining the eligibility window, for the case of $\alpha = 0.05$. If an interim *p* value falls between these cutoffs, the result is considered "inconclusive" or "promising", and sample size is incremented, subject to some finite cap on the sample size. If an interim *p* value falls below the lower cutoff, sampling terminates with a decision of "significant". If an interim *p* value falls above the upper cutoff, sampling terminates with a decision of "nonsignificant" (fail to reject the null) or "futile". The case of $w = 0$ is equivalent to a fixed-N sampling procedure. Values of $w \leq 1$ are posited to be representative of informal heuristic decisions used by some researchers. Egregious N-hacking ($w > 2$, red) is incompatible with reporting p values.
