# Supplementary material for: Is N-Hacking Ever OK? The consequences of collecting more data in pursuit of statistical significance
Source: PLoS Biol. 2023 Nov 1;21(11):e3002345. doi: 10.1371/journal.pbio.3002345 (PMC10619921; doi:10.1371/journal.pbio.3002345)
Supplement: S2 Table — (PDF) [file pbio.3002345.s002.pdf]

Procedure	<N>	FP_0	Power	PPV
A. Fixed-N	8	0.05	0.46	0.51
B. Augmented	9	0.12	0.78	0.42
C. Fixed-N	9	0.12	0.68	0.39
D. Fixed-N	9	0.19	0.78	0.31

S2 Table. N-hacking compared with alternative fixed-N policies. **A.** Performance of a fixed-N procedure with $N = 8,\ \alpha = 0.05$. **B.** Performance characteristics of a constrained sample augmentation condition that had higher power but lower PPV: $N_{init} = 8, \alpha = 0.05, w = 5$. **C.** Fixed-N procedure where N and α are chosen to match the final sample size <N> (blue) and false positive rate FP_0 (yellow) of the procedure in B, showing that this is worse than B (lower power and PPV). **D.** Fixed-N procedure where N and α are chosen to match <N> and power (green) of procedure B, showing that this is worse than B (higher FP_0 and lower PPV).
