# Supplementary material for: Is N-Hacking Ever OK? The consequences of collecting more data in pursuit of statistical significance
Source: PLoS Biol. 2023 Nov 1;21(11):e3002345. doi: 10.1371/journal.pbio.3002345 (PMC10619921; doi:10.1371/journal.pbio.3002345)
Supplement: S1 Fig — (PDF) [file pbio.3002345.s003.pdf]

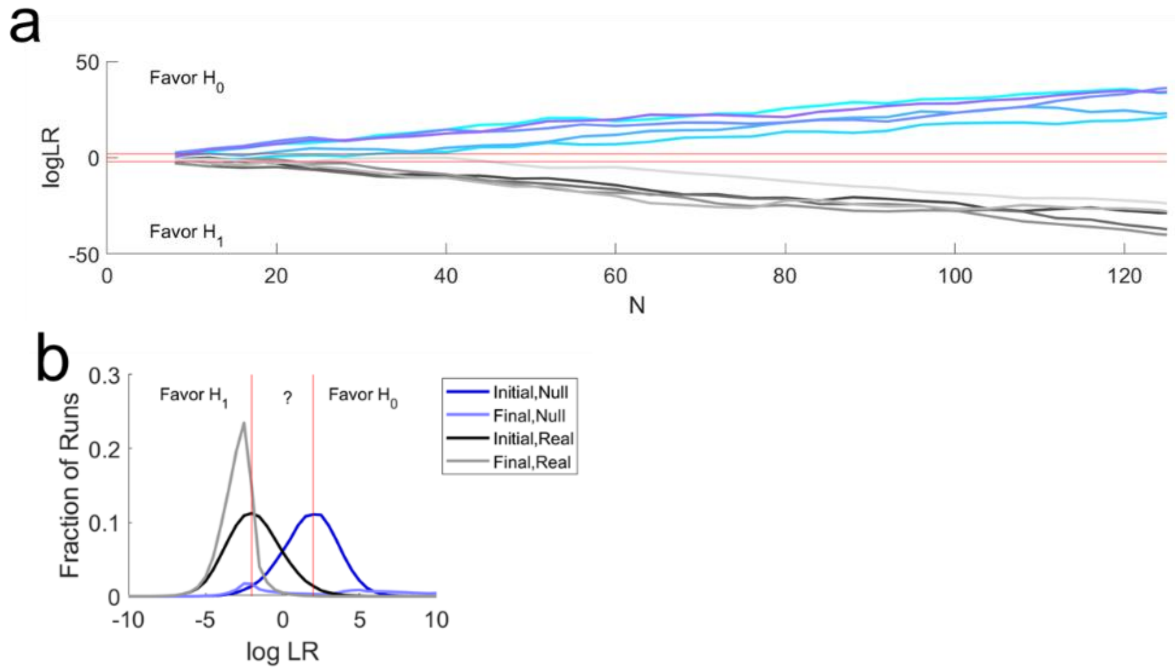

S1 Fig. Data from the simulations shown in Fig 1, re-analyzed using log likelihood ratios instead of *p* values. Here the null hypothesis (H_0, no effect) is compared to a specific alternative hypothesis (H_1, a positive effect of 1SD). The weight of evidence is measured by the $\log_{10}$ of the Likelihood Ratio (log LR) of the two hypotheses. A negative number indicates more evidence for the alternative (a real effect) and a positive number indicates more evidence for the null (no effect). A possible decision criterion is indicated by the red lines: accept the null hypothesis if $\log LR > 2$ (i.e., likelihood ratio 100:1 in favor of null), accept the alternative if $\log LR < -2$ (100:1 in favor of alternative), and consider the study inconclusive if log LR is between those thresholds. **a.** Evolution of log LR with sample growth, for five example runs from the egregious N-hacking simulation analyzed in Fig 1, in which the null hypothesis was true (shades of blue). Five example runs from experiments with a real effect of 1SD (shades of gray) are also shown. As the sample size increases, log LR fluctuates. But unlike the *p* value, which fluctuates randomly with no net trend, log LR *trends systematically* toward the null conclusion for null experiments, and systematically toward the real-effect conclusion for experiments with real effects. This matches experimentalists' intuition that having more data is always more informative. **b.** Distributions of final log LR values after egregious N-hacking (c.f. Fig 1b, distribution of *p* values of the same runs). For the null experiments, the vast majority of inconclusive cases (dark blue curve, area between the red lines) resolve to *negative* results (most of the area under the pale blue curve is shifted off the scale to the right). For the experiments with real effects, most of the inconclusive cases (black curve, area between the red lines) resolve to positive results (most of light gray curve is shifted to the left of the criterion for accepting H_1). It is controversial whether or not Bayes Factors are immune to N-hacking [S1 Fig references 1-4], however.

S1 Fig References

1. Yu EC, Sprenger AM, Thomas RP, Dougherty MR. When decision heuristics and science collide. Psychon B Rev. 2014;21(2)(2):268-82. PubMed PMID: CCC:000334174700003.
2. de Heide R, Grunwald PD. Why optional stopping can be a problem for Bayesians. Psychon B Rev. 2021;28(3)(3):795-812. PubMed PMID: CCC:000590490300001.
3. Rouder JN. Optional stopping: No problem for Bayesians. Psychon B Rev. 2014;21(2)(2):301-8. PubMed PMID: CCC:000334174700005.
4. Anderson RB, Crawford JC, Bailey MH. Biasing the input: A yoked-scientist demonstration of the distorting effects of optional stopping on Bayesian inference. Behavior Research Methods. 2022;54(3)(3):1131-47. PubMed PMID: CCC:000693474600001.
