# Supplementary material for: Is N-Hacking Ever OK? The consequences of collecting more data in pursuit of statistical significance
Source: PLoS Biol. 2023 Nov 1;21(11):e3002345. doi: 10.1371/journal.pbio.3002345 (PMC10619921; doi:10.1371/journal.pbio.3002345)
Supplement: S1 Appendix — (PDF) [file pbio.3002345.s004.pdf]

S1 Appendix. Definitions of terms and variables as used in this paper

Sample	A group of representatives randomly selected from a larger population and intended to represent it
Observation, data point	One of the individuals or representatives in a sample
H_0 Null hypothesis (no effect)	For example, in an independent sample *t*-test comparing samples from populations A and B, the null hypothesis is that the means of the groups are the same: $H_0: \mu_A = \mu_B$
H_1 Alternative hypothesis (effect)	For the *t*-test example, the alternative is that means of the populations are not the same: $H_1: \mu_A \neq \mu_B$
N Sample size	In a fixed-N procedure: the number of observations in each group<br>In an incrementing procedure:<br>N_{init} Initial sample size<br>N_{incr} Number of observations added each time<br>N_{max} Maximum sample size before stopping
p Value returned by statistical null hypothesis test	The fraction of such experiments in which one would observe a difference at least as great as the observed difference, if in fact H_0 were true.
α Significance criterion	A criterion to reject H_0 only if $p < \alpha$
w Eligibility window	In the N-increasing procedure, defines how close to α a p value must be to collect more data as follows:<br>$\alpha \leq p < (1 + w)\,\alpha$
FP_0 False Positive Rate on the Null (Type I Error Rate)	For any procedure, probability of rejecting the null if the null is true: $FP_0 \equiv P(reject\ H_0 \vert H_0)$<br>For the fixed-N case in this paper, $FP_0 \equiv \alpha$<br>In simulations: the observed frequency of positive results when both samples drawn from the same distribution.
E Effect size	The true difference in the means of the two populations being compared, expressed in standard deviations:<br>$E \equiv \frac{\vert\mu_A - \mu_B\vert}{\sigma}$
$P(H_1)$ Prior probability of an effect	The probability H_0 is false, before considering the data. In simulations: fraction of experiments in which the samples were drawn from distributions with different means.
Power	The probability that a real difference will be found to be significant: $Power \equiv P(reject\ H_0 \vert H_1)$<br>Depends on α, N and effect size E
PPV Positive Predictive Value	The probability that an effect that was deemed significant is in fact real: $PPV \equiv \mathrm{P}(H_1 \vert\, reject\ H_0)$<br>Depends on α, Power, and prior $P(H_1)$<br>Related to False Positive Risk: $FPR = 1 - PPV$
