# Supplementary material for: Is N-Hacking Ever OK? The consequences of collecting more data in pursuit of statistical significance
Source: PLoS Biol. 2023 Nov 1;21(11):e3002345. doi: 10.1371/journal.pbio.3002345 (PMC10619921; doi:10.1371/journal.pbio.3002345)
Supplement: S2 Appendix — (PDF) [file pbio.3002345.s005.pdf]

S2 Appendix. A conservative bound on Type I Error Rate

In some cases a strictly confirmatory study is needed to inform a high-stakes binary decision, in which case a prespecified sampling procedure is essential. In such cases researchers may still feel that pre-registering a fixed sample size is overly constraining, as they want the flexibility to abandon data collection early if results are not promising, or to continue data collection if results are very promising. Although there are many standard adaptive sampling procedures available, many researchers find them prohibitively complicated or confusing. Therefore it might be of some value to point out that the procedure described, if pre-registered, would be entirely valid in a confirmatory setting.

Although no formal proof has been provided, the simulated data strongly suggest that if one committed to the simulated decision rule formally in advance – without any multiple comparison correction for re-tests, as simulated – the following inequalities apply (dotted lines, Figure 5e):

$FP_0 < \alpha(1 + \frac{w}{2})$ for $N_{incr} \leq N_{init}$

$FP_0 < \alpha(1 + \frac{w}{4})$ for $N_{incr} = N_{init}$

These appear to be loose bounds; in many conditions the false positive rate falls well below this value. But they have the virtue of being trivial to calculate. For example: an N-increasing procedure with $w = 0.4$, $N_{init} = 10$, $N_{incr} = 10$, $N_{max} = 50$, would have a bound of $FP_0 < 0.0550$ by rule of thumb, compared to the simulation result of $FP_0 = 0.0541 \pm 0.0001$ (mean ± SD). The code provided [13] can be used to numerically estimate the false positive rate for any parameter combination. Using worst-case parameters $\alpha = 0.10$ $N_{incr} = 1$ $w = 9$ for $N_{init} = 128$ with $N_{max} = 256$ still did not exceed this empirical bound, despite allowing for up to 128 "peeks" at the data with no peeking penalty.

Thus, if one committed to using the procedure simulated in Fig 2A ($\alpha = 0.05$, $w = 1$) one could conservatively report $\alpha_{procedure} < 0.075$ regardless of N_{init} and N_{incr}. Or one could choose a nominal $\alpha = 0.0\overline{33}, w = 1$ ($p_{max} = 0.0\overline{66}$) in the procedure to guarantee $\alpha_{procedure} < 0.05$. Or if a lab followed a general policy of collecting more data only if $p < 2\alpha$ ($w = 1$), they could conservatively correct for the possibility of unplanned sample augmentation by reporting an adjusted false positive rate of 1.5α (for all their experiments, whether or not augmentation occurred in that case).

I have not done a quantitative comparison of the performance characteristics of this procedure to well-established adaptive sampling procedures, and I am making no claim that it would be especially efficient or optimal. I have not found this exact sampling procedure described in the statistical literature, although new adaptive sampling procedures are continually being proposed, and the one explored here belongs in the generally family of promising zone methods.
